# Toward Fully Automated Personalized Orthopedic Treatments: Innovations and Interdisciplinary Gaps

**DOI:** 10.3390/bioengineering11080817

**Published:** 2024-08-12

**Authors:** Yunhua Luo

**Affiliations:** 1 Department of Mechanical Engineering, University of Manitoba, Winnipeg, MB R3T 2N2, Canada; yunhua.luo@umanitoba.ca; 2 Biomedical Engineering (Graduate Program), University of Manitoba, Winnipeg, MB R3T 2N2, Canada

**Keywords:** bone fracture and defect, personalized device and treatment, scaffold, implant, emerging technologies, integration, automation, interdisciplinary gaps

## Abstract

Personalized orthopedic devices are increasingly favored for their potential to enhance long-term treatment success. Despite significant advancements across various disciplines, the seamless integration and full automation of personalized orthopedic treatments remain elusive. This paper identifies key interdisciplinary gaps in integrating and automating advanced technologies for personalized orthopedic treatment. It begins by outlining the standard clinical practices in orthopedic treatments and the extent of personalization achievable. The paper then explores recent innovations in artificial intelligence, biomaterials, genomic and proteomic analyses, lab-on-a-chip, medical imaging, image-based biomechanical finite element modeling, biomimicry, 3D printing and bioprinting, and implantable sensors, emphasizing their contributions to personalized treatments. Tentative strategies or solutions are proposed to address the interdisciplinary gaps by utilizing innovative technologies. The key findings highlight the need for the non-invasive quantitative assessment of bone quality, patient-specific biocompatibility, and device designs that address individual biological and mechanical conditions. This comprehensive review underscores the transformative potential of these technologies and the importance of multidisciplinary collaboration to integrate and automate them into a cohesive, intelligent system for personalized orthopedic treatments.

## 1. Introduction

Bone fractures and defects are global health concerns, particularly among the elderly, often resulting from conditions like osteoporosis, diabetes, arthritis, and bone cancer. A recent global study by the Bill & Melinda Gates Foundation reported 455 million cases with acute or long-term symptoms [1]. In Canada, osteoporosis alone can cause about 30,000 fractures annually [2]. In the treatment of fractured or diseased bone tissues, conventional methods such as autografts, allografts, and xenografts encounter limitations due to their restricted availability, timing constraints, and potential risk of biological incompatibility [3]. Orthopedic devices, encompassing both scaffolds and implants, provide the benefits of mass production and immediate availability. It is crucial to distinguish between the intended purposes of scaffold and implant treatments. The primary purpose of a scaffold is to provide a temporary structure that can support and guide the growth of new tissue. It is designed to degrade over time, allowing the body’s natural tissue to replace it. Scaffolds are commonly used in tissue engineering, where the aim is to regenerate lost or damaged tissue. An implant is a medical device meant to replace, support, or act as a missing biological structure. Unlike scaffolds, they are often designed to be permanent, though some can be removed later.

Currently, off-the-shelf orthopedic devices are prevalent in clinical settings, primarily for their cost-effectiveness and immediate accessibility. However, their long-term success rates have proven to be disappointing [4,5,6,7,8], especially among younger patients [8,9,10]. This can be attributed to the diverse and unique conditions presented by each patient [11,12,13], including overall health status, bone quality, size, and shape at the affected site, and distinct biological and mechanical characteristics, which generic devices often fail to address comprehensively. While not yet entirely personalized, customized devices demonstrate significant potential in enhancing long-term outcomes [14,15,16]. Recent technological advancements from diverse disciplines strongly push toward personalized orthopedic treatments, as explored later in this paper. Nevertheless, these advancements have yet to be seamlessly integrated and fully automated for use by clinicians. Consequently, crafting personalized devices poses challenges, either necessitating interdisciplinary expertise for clinicians or incurring significant costs and time when convening a multidisciplinary team for each patient. Moreover, notable interdisciplinary gaps and challenges persist [17], impeding the seamless integration of workflows for personalization.

The aim of this paper is to identify the primary interdisciplinary gaps and propose strategies to bridge them, thereby paving the way for a seamless, fully automated end-to-end personalization workflow for orthopedic treatments in the foreseeable future. In the subsequent sections, we first provide an overview of the key stages and standard practices in the clinical lifecycle of orthopedic treatments and then spotlight the cutting-edge technological advancements essential to personalizing these treatments, followed by a discussion on the primary interdisciplinary gaps and possible solutions. We wish to highlight that, due to the extensive scope of this review, the volume of pertinent references is considerable. Consequently, it was not feasible to include all of them in this paper. Instead, we have chosen to include only the most representative and recent references.

## 2. The Lifecycle of Standard Orthopedic Treatments

**Figure 1:**
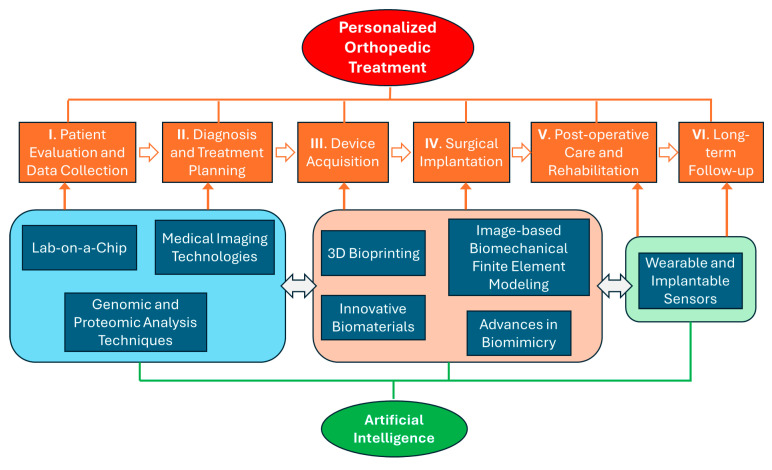
The existing lifecycle of orthopedic treatments and the integration of innovative technologies for personalization.

Although a scaffold and an implant serve distinct treatment purposes, they share common stages in their lifecycles. Figure 1 visually depicts the primary stages in the standard lifecycle of scaffold and implant treatments. The subsequent subsections outline the standard practices in these stages, emphasizing the extent of personalization. Both the scaffold and implant will be referred to as an “orthopedic device” unless differentiation is necessary. This is a general overview; the exact stages and steps may differ depending on the medical facility, treatment type, patient condition, and advancements in the field.

I. Patient Evaluation and Data Collection

The initial stage of orthopedic treatment revolves around meticulous data collection about the patient. This includes recording fundamental physiological parameters such as age, weight, and height. An in-depth medical history, noting past surgical interventions, known allergies, current medications, and relevant genetic information, is compiled. Laboratory tests, encompassing blood and urine samples, are performed to gauge the patient’s overall health and pinpoint specific health markers. Conducting tests to identify potential allergies, especially those tied to materials in the orthopedic device, is paramount. Understanding the patient’s lifestyle, daily activities, and occupation, as well as their expectations regarding recovery and potential risks related to the treatment, plays a pivotal role in tailoring the therapeutic approach. The integrity of the affected bone stands as a central consideration in devising the treatment plan. Techniques such as X-rays, MRI (Magnetic Resonance Imaging), and CT (Computed Tomography) scans are employed to obtain high-resolution images of the affected region. Gathering exhaustive information on conditions that might compromise bone quality—like osteoporosis, rheumatoid arthritis, and diabetes—is of utmost importance. Some conditions may introduce complications in the treatment or even be the primary culprits behind bone fractures. If tumors are suspected, biopsies are carefully examined. Additionally, it is vital to assess any medications the patient might be taking that could adversely impact bone health.

Ensuring the accuracy and comprehensiveness of patient data gathered at this juncture is pivotal for the success of subsequent treatment planning.

II. Diagnosis and Treatment Planning

The stage of diagnosis and treatment planning is both critical and complex, underpinning the foundation for personalized treatment. Adherence to best practices is paramount, and precision in decision-making becomes essential to tailor the most appropriate intervention for each patient. Leveraging insights from the previous evaluations, a thorough diagnosis is conducted. The data collected in stage I are carefully analyzed to assess the suitability of a scaffold or implant for the patient. At this point, an interdisciplinary team of radiologists, biomaterial scientists, biomechanical engineers, prosthetists, and orthopedic surgeons converge, leveraging their combined expertise to determine the most fitting implant or scaffold. Historical cases and experiences usually serve as valuable references during the treatment planning process. Once a device type has been agreed upon, the team must decide between off-the-shelf and custom-made solutions, a quintessential and pivotal decision in this phase.

III. Device Acquisition

To obtain the chosen orthopedic device, practitioners can opt for either off-the-shelf procurement or customized design/fabrication. While they represent contrasting approaches, both rely on similar deciding factors, encompassing patient demographics, material biocompatibility, biomechanical demands, and economic factors. The foundational data from stage I primarily guide the technical choices at this stage. The device’s dimensions and form should be congruent with the patient’s anatomy. Conditions like osteoarthritis, bone tumors, or fractures affect the device selection. A patient’s health, age, and lifestyle are pivotal. For instance, younger, active individuals often necessitate robust devices capable of enduring intense activities. The material’s long-term impact is foremost. Materials vary in their biological interactions, and some might trigger allergies or adverse reactions in patients. This choice is vital for ensuring a harmonious long-term relationship between the device and adjacent tissue. Devices should cater to specific biomechanical needs. Devices meant for weight-bearing bones differ from those for non-weight-bearing sections. Similarly, dynamic areas like joints demand different specifications compared to static zones. Economic factors, including insurance provisions and a patient’s financial capacity, also play a role in device selection.

The primary difference between the two acquisition methods is the depth of personalization. While personalization is present even in off-the-shelf options, it is substantially more pronounced in customized alternatives. Nevertheless, today’s customized designs have yet to fully harness the potential offered by recent technological innovations, which will be discussed later in this paper.

IV. Surgical Implantation

The primary objective of surgical implantation is to secure the device within the body while minimizing potential harm. The complexity of this procedure varies based on the device’s intended function and its anatomical location. One primary concern is the forces acting upon the device and adjacent bones during insertion. Inappropriate or excessive forces can compromise the device’s integrity or even induce fractures in the surrounding bone, jeopardizing both immediate and long-term outcomes. It is crucial that the design of the device comes with explicit guidelines on implantation to mitigate these risks. The specifics of the forces—both in type and magnitude—often depend on the device’s particular design and its intended position within the body. During insertion, whether the surgeon applies force manually or uses specialized equipment, care is essential. This is particularly significant for press-fit implants that stabilize via a tight interference fit. A surgeon, relying on their skill and experience, may need to delicately adjust, rotate, or maneuver the device to achieve optimal positioning, and each of these manipulations introduces forces. Some orthopedic surgeries, like hip joint replacements, require impactful seating of the implant components, sometimes necessitating the use of a mallet. When implants anchor to the bone, preparation tools like drills or reamers come into play, each introducing significant forces. Furthermore, securing the device using sutures, screws, pins, or other hardware inherently applies force. Beyond direct forces, other considerations also come into play. For instance, during many procedures, soft tissues (like muscles or tendons) are retracted to enhance visibility. While necessary, this can indirectly exert force on the implant or its adjacent structures. Temperature fluctuations, such as those from heat during bone cement polymerization, can cause expansion or contraction, placing stress on the device. Post-implantation, the body’s natural inflammatory response may cause swelling, potentially exerting additional pressure on the device. Therefore, the surgical placement of devices demands a deep understanding of the involved forces and a precise, skilled approach to ensure successful outcomes, especially as these forces can vary based on the mechanical properties of the affected bone, the specific device, and the procedure.

V. Post-Operative Care and Rehabilitation

Post-operative care and rehabilitation are not mere afterthoughts; they are crucial determinants of the success of orthopedic treatments. The difference between an orthopedic device that elevates one’s quality of life and one that underperforms, possibly requiring removal or revision, frequently lies in the quality of post-operative care and systematic rehabilitation. Proper post-operative care is essential for the device to integrate seamlessly with neighboring tissues. Consider bone implants: diligent post-operative attention can promote osseointegration, where the bone grows around and securely anchors the implant. Consistent monitoring after the procedure is also pivotal to detect and address potential issues early on, be it infections, the instability of the device, or unfavorable reactions.

Rehabilitation, with a particular emphasis on physiotherapy, is vital for restoring functionality. It aids in enhancing the range of motion, strengthening muscles, and honing coordination around the implanted site. This is particularly essential in cases of joint replacements, where the primary objective is to recover lost mobility. However, the forces exerted during physiotherapy require careful attention. While tissues necessitate proper stress stimulation for regeneration and growth, undue stress could risk fracturing the device or adjacent bones or potentially lead to a separation between the device and bone. Given the individualized nature of each patient’s treatment, customizing physiotherapy is vital for efficient and swift rehabilitation, yet standard procedures are often employed. Building on the premise of tailored treatments, it is essential to empower patients with thorough knowledge. This encompasses understanding activity limitations, recognizing early signs of potential complications, and mastering the proper care for the surgical site. Such informed patients can actively contribute to and enhance their recovery trajectory.

VI. Long-Term Follow-Up

Despite its demanding nature, long-term follow-up is essential in orthopedic treatments, extending beyond the immediate priorities of patient safety and well-being. Long-term follow-up is a vital avenue for gathering data that will improve future orthopedic treatments. As the body adapts to the device, continuous monitoring is essential for ensuring its ongoing integration, such as osseointegration and overall stability. Some complications may evade detection in the immediate post-operative period. Issues like implant loosening, adverse tissue reactions, or latent infections can surface months or even years post-surgery. Implants, especially those under frequent biomechanical stress like joint replacements, are prone to wear. Regular check-ups help spot and address such concerns early on. For bioactive scaffolds, consistent monitoring assesses the progress of tissue regeneration and its integration. Long-term outcome observations equip medical professionals with insights to optimize device selection and rehabilitation protocols, promoting better recovery and functionality for future recipients. Feedback from extended follow-ups is invaluable to biomedical engineers and researchers. It guides them in refining device designs based on real-world performance and patient experiences. Beyond just assessing the device’s mechanical integrity, it is vital to gauge its broader impact on a patient’s quality of life, which includes pain levels, mobility, and overall satisfaction.

## 3. Innovative Technologies Driving Personalization of Orthopedic Treatments

Emerging technologies from a range of disciplines are driving the evolution of personalized orthopedic treatments. In this section, we explore the most influential ones among these technologies: artificial intelligence (AI), innovative biomaterials, cutting-edge genomic and proteomic techniques, novel lab-on-a-chip, sophisticated medical imaging, 3D printing and bioprinting, image-based biomechanical finite element modeling, innovations in biomimicry, and the introduction of wearable and implantable sensors. Figure 1 illustrates how these innovative technologies can be integrated into the existing life cycle of orthopedic treatments. We will delve into how these technological strides are transforming and enhancing personalized treatment methodologies across the previously discussed treatment stages.

### 3.1. Artificial Intelligence (AI)

Artificial intelligence (AI), and more specifically, its subsets, machine learning (ML) and deep learning (DL), is revolutionizing healthcare. While still in its nascent stages, its potential is already evident in markedly improving the efficiency and accuracy of medical data interpretation. This ongoing transformation promises to expedite patient care and streamline healthcare delivery in the near future [18]. In the realm of orthopedic treatments, AI’s influence is multifaceted. It can illuminate the intricacies of healing mechanisms [19], streamline the discovery of innovative scaffold materials [20,21], optimize biomechanical models [22], enhance 3D printing configurations [23], facilitate advanced data analysis [24], and push the boundaries in scaffold [25] and implant design [26]. Consider the traditionally cumbersome process of developing new materials for medical applications: deep learning can transform this from a time-intensive, trial-and-error undertaking to an efficient and systematic progression [21]. Similarly, while determining optimal 3D printing settings for innovative materials has historically been resource-intensive, ML algorithms now enable swift and precise configurations, upholding the integrity of the finished product [23]. Taking the intricate anatomy of the shoulder as a case in point, surgical planning can be arduous. Conventionally, surgeons rely on X-rays, which can be time-intensive and susceptible to errors. Deep learning can enrich the process, fine-tuning X-ray analyses to distinguish between shoulder implant types and enhance surgical precision [22]. Further, 3D convolutional neural networks (3D CNNs), once trained with specific digital blueprints, are able to excel in predicting the robustness and functionality of innovative scaffold designs, positioning AI at the vanguard of design evolution in tissue engineering [9]. In the broader market, myriad implant designs abound, yet discerning the optimal configuration remains challenging. Merging ML with intricate analytical methodologies offers nuanced insights, refining designs such as hip implants to amplify patient comfort and efficacy [26].

While AI’s current impact on orthopedic treatment personalization is undoubtedly transformative, it is only scratching the surface. The potential of AI in reshaping orthopedic treatments is vast, presenting an array of yet-to-be-unveiled challenges and opportunities. It is important to note that AI algorithms require high-quality and comprehensive datasets to function effectively. Data inconsistencies or insufficient datasets can compromise the accuracy of AI predictions. The availability of robust and diverse datasets is crucial for maximizing the potential of AI in personalized orthopedic treatments.

### 3.2. Innovative Biomaterials

Innovative biomaterials have greatly advanced regenerative medicine, including orthopedic treatments [27,28]. Materials such as polycaprolactone (PCL), polylactic acid (PLA), and polyglycolic acid (PGA) have gained popularity due to their biodegradable nature, allowing them to degrade over time and be replaced by the patient’s own tissues [29,30]. The degradation rate of such material can be adjusted by modifying its chemical composition, allowing it to synchronize with the patient’s healing capacity. Functionally graded materials (FGMs) are composite materials where the composition and the properties vary spatially, effectively mimicking the natural gradation present in the affected bone [31,32,33]. Bioactive glasses are known for their osteoconductive properties [34,35], making them ideal for patient-specific bone regenerative applications. They bond well with bone and can stimulate the formation of new bone tissue. Hydrogels are water-swollen polymers that can mimic the extracellular matrix of many tissues and have been widely studied for applications in soft tissue engineering [36,37,38]. Shape memory alloys can return to a predefined shape upon heating, which can be especially useful for creating implants tailored for minimally invasive procedures or adapting them to a patient’s unique anatomy [39,40]. Recent nanomaterials can enhance mechanical properties, biodegradability, and personalized drug delivery capabilities. For instance, hydrogenated silicene nanosheet-functionalized scaffolds have shown promise in enabling immuno-bone remodeling, offering a novel approach to bone regeneration. These nanomaterials provide a conducive environment for cell proliferation and differentiation, significantly improving the outcomes of orthopedic treatments [41,42,43]. Surface modification techniques like plasma treatment, chemical grafting, or layer-by-layer deposition can enhance the surface properties of implants [44,45,46]. These techniques ensure better cell adhesion, proliferation, and differentiation, which can be critical for patient-specific treatments. The use of decellularized extracellular matrix, collagen, silk, and other naturally derived materials offers an environment that can be more conducive to tissue regeneration compared to entirely synthetic scaffolds [47,48,49]. Smart and stimuli-responsive materials can change their properties in response to external stimuli such as temperature, pH, or electrical signals [50,51]. Such responsiveness can be harnessed for controlled drug release or dynamic structural support tailored to individual needs. Combining synthetic and natural materials can cater to both structural requirements and biological compatibility [52,53]. This ensures that the orthopedic device not only supports the damaged area but also provides an environment conducive to natural healing [54].

These cutting-edge biomaterials and techniques pave the way for highly personalized, biocompatible, and multifaceted solutions, addressing the intricacies of tissue regeneration and replacement.

### 3.3. Genomic and Proteomic Analysis Techniques

Genomic analysis involves the study of an organism’s entire genetic code or genome [55]. It examines the DNA sequence to understand its structure, function, and evolution. This includes identifying genes, mutations, and other genetic variations. Proteomics is the large-scale study of proteins, particularly their structures and functions. Proteins are vital parts of living organisms, responsible for the majority of the molecular functions in every life form. Proteomic analysis can help identify and quantify the proteins in a sample and understand their interactions, post-translational modifications, and more.

Genomic and proteomic analyses can give insights into how an individual might react to certain materials, thereby guiding the selection of biomaterials for orthopedic devices [56,57,58]. For example, if an individual’s genomic data suggest a predisposition to metal allergies, an alternative material can be chosen for their implant. For orthopedic devices that deliver therapeutic agents, genomic and proteomic data can guide which agents might be most effective for a particular patient [59,60]. Single-cell techniques, such as single-cell transcriptomics, have revolutionized tissue engineering and regenerative medicine by allowing the detailed characterization of cellular heterogeneity within tissues [55,60]. Moreover, genomic analysis can help in predicting potential adverse reactions, ensuring that the device material is compatible with the patient’s biology. This approach can significantly enhance the safety and efficacy of orthopedic devices [60,61,62]. This is especially relevant in cases where the implant is designed for localized drug delivery, such as in tumor treatments. By analyzing an individual’s genomic and proteomic data, clinicians can predict potential adverse reactions, ensuring that the device material is compatible with the patient’s biology [61]. Genomic data can provide insights into a patient’s tissue regeneration capabilities [62]. For bioactive scaffolds that aim to encourage tissue growth, understanding these capabilities at a molecular level can guide the scaffold’s design to better integrate with the patient’s tissues. Genomic and proteomic analyses can provide a deeper understanding of the underlying causes of a patient’s condition, such as bone degeneration or cartilage loss [63,64]. This understanding can inform the design and functionality of orthopedic devices to address the root cause, rather than just the symptoms. After implantation, changes in the local genomic or proteomic profile can indicate potential complications, infections, or adverse reactions. This could lead to earlier interventions and improved outcomes [65]. Proteomic analyses further provide a deeper understanding of protein interactions and modifications, crucial for optimizing the biocompatibility and functionality of the implants [58,65]. With the growing field of precision medicine, where treatments are customized to individual patients based on their unique genetic, environmental, and lifestyle factors, genomic and proteomic analyses play a crucial role. In the context of orthopedic devices, this could mean designing treatments tailored to each patient’s molecular profile, optimizing efficacy and reducing potential risks.

Genomic and proteomic analyses offer a deeper, molecular-level understanding of a patient’s biology. When applied to the field of personalized orthopedic treatments, they provide the opportunity for more tailored, effective, and safer interventions, truly personalizing the approach to individual patient needs.

### 3.4. Lab-on-a-Chip

Lab-on-a-chip (LOC) technologies, often referred to as μ-TAS (micro-total analytical systems), are transformative tools for the immediate and accurate analysis of real-world samples using intricately engineered microdevices [66,67]. Their potential to revolutionize automation and customization in medical therapies, including orthopedic treatments, is profound [68,69,70]. When compared with traditional lab-based testing, the merits of LOC technologies become evident in several ways. LOC devices can process and yield results from samples in a fraction of the time—often within mere minutes or hours, as opposed to the prolonged durations associated with many standard lab tests. The microenvironment within LOC systems facilitates meticulous control over reactions, often leading to superior test precision and accuracy [71]. Such expediency and accuracy can be crucial for assessing the state of an injury or determining the best treatment approach. LOC systems can integrate various testing steps into a single device, automating processes that would typically require manual intervention in a traditional lab. This automation reduces the risk of human error and streamlines the diagnostic process. LOC platforms can meet personalized patient needs, whether it is through cell culture, drug-response analysis, or biomechanical evaluations. Such tailored assessments can equip clinicians with invaluable insights to design individualized treatment plans. Given their microscale nature, LOC technologies necessitate significantly less sample material. This is especially advantageous when drawing samples from restricted sources like joint fluids or bone marrow. Over time and with scale, LOC devices can reduce the cost per test because of reduced reagent use, fewer personnel requirements, and less infrastructure compared to traditional labs.

In the realm of orthopedic treatments, LOC technologies can undertake a multitude of specialized tasks. In the phase of diagnosis and initial assessment, LOC can swiftly detect biomarkers in the blood, urine, serum, or synovial fluid indicative of bone or joint diseases, inflammation, or healing processes [72]. Genomic or transcriptomic analyses using LOC systems can pinpoint genetic predispositions or molecular pathways relevant to orthopedic conditions [73,74], aiding in treatment strategy formulation. Emerging LOC technologies aim to gauge bone quality attributes such as density or mineralization [75], although miniaturization remains challenging. LOC can determine specific ion, nutrient, or metabolite levels in the blood or joint fluid [76], signaling bone health or a disease state. Understanding the rheological properties of synovial fluid using LOC can provide insights into joint health, especially in conditions like osteoarthritis.

For orthopedic treatment planning and personalization, LOC devices can culture patient-derived cells, such as chondrocytes or osteoblasts [77]. Observing how these cells react to various materials on-chip can guide the selection of material so that it has the best biocompatibility with the patient. LOC devices can ascertain how patient-specific cells or tissues react to different drugs [78], pinpointing the most effective therapeutic approaches. Some LOC devices can evaluate the mechanical attributes of tissues or cells [79], instrumental in understanding bone or cartilage health.

In the stage of post-treatment monitoring and follow-up, some LOC devices encompass imaging capabilities, enabling the real-time observation of cell behavior, tissue regeneration, or drug responses [71]. Given the concerns about post-surgical infections in orthopedics, LOC devices can swiftly identify bacteria or other pathogens in fluid samples [73,80], enabling prompt intervention.

As these LOC technologies advance, they promise to augment their value in personalized orthopedic treatments, furnishing clinicians with an ever-expanding toolkit to assess patient needs and customize interventions.

### 3.5. Medical Imaging Technologies

Medical imaging technologies play a pivotal role in personalizing orthopedic treatments throughout the entire lifecycle. In the pre-operative planning stage, high-resolution imaging, such as MRI or CT scans, provides detailed insights into a patient’s unique anatomy. This detailed visualization facilitates the selection of orthopedic devices that better match individual anatomical structures [81]. Advanced imaging can detect abnormalities or changes in the affected bones [82,83], which helps in tailoring treatments to address specific pathological conditions. In the design stage, image-based finite element modeling (FEM) has emerged as a powerful tool in the design of personalized orthopedic devices [84,85,86], which will be reviewed later in this section. In the stage of implantation, real-time imaging, like intraoperative MRI or CT, can guide surgeons during the implantation process. This ensures the accurate placement and integration of the device with surrounding tissues. In the post-operative care stage, imaging plays pivotal roles in monitoring the osseointegration of orthopedic devices with the surrounding bones [87] and identifying early signs of potential issues like infections, implant migration, tissue necrosis, or other complications, enabling timely interventions [88]. Especially relevant for bioactive scaffolds designed to support tissue regeneration, imaging techniques can monitor the progress and health of regenerating tissues over time [89,90,91]. For implants designed to release therapeutic agents or drugs over extended periods, imaging can monitor the distribution, release rate, and effectiveness of these agents in the targeted tissues [92,93]. Some scaffolds are designed to biodegrade over time. Advanced imaging can track this degradation process, observing how the body responds and how natural tissues take over the space and function of the degrading scaffold [94,95,96]. For implants, especially those in weight-bearing or high-motion areas, imaging can detect early signs of wear or potential failures [97,98], guiding decisions about possible future interventions or replacements. Medical imaging is invaluable in research settings, allowing for standardized comparisons between different implant designs or materials, the assessment of their interaction with biological tissues, and the measurement of their performance over time.

### 3.6. Image-Based Biomechanical Finite Element Modeling

Image-based biomechanical finite element modeling (IB-Bio FEM) utilizes patients’ medical images, such as CT or MRI, to construct patient-specific dynamics and finite element models [99,100]. IB-Bio FEM is pivotal for personalized design and assessment of orthopedic devices. Central to the personalized design and evaluation of orthopedic devices, IB-Bio FEM sources vital information directly or indirectly from a patient’s medical images. These data encompass the geometric shape of the compromised bone and its density, quality, and mechanical properties. Geometric information of the affected bone can guide the device design for an optimal fit to the defect [101,102,103]. Bone density derived from the patient’s CT scans can be used to estimate bone mechanical properties such as Young’s modulus and yield stress [104,105,106,107]. These properties have multiple uses. They guide the selection of device material of appropriate stiffness to prevent stress shielding, a primary factor in long-term failure. A multibody dynamics model can be constructed with the properties to simulate physiological forces exerted on the affected bone [108], which are essential for designing the device. A finite element model integrated with the properties can simulate stress and strain distributions within the device and in the surrounding bones [109,110,111,112,113], thereby pinpointing potential zones of failure or adverse tissue responses.

Before actual implantation, IB-Bio FEM can virtually test the mechanical strength and performance of the device, ensuring its suitability for the patient’s specific mechanical needs [114]. IB-Bio FEM allows for the design of devices with functionally graded properties [115,116,117], where the mechanical properties vary in a similar way as in the affected bone. This can lead to more natural integration and further reduce stress shielding. By integrating IB-Bio FEM with computational wear, fatigue, and degradation models, it is possible to predict the long-term performance of the device [118,119]. This is invaluable for ensuring the longevity of the implant. Medical professionals can use IB-Bio FEM predictions to make informed decisions regarding the choice of implant, surgical approach, and post-operative care [120,121,122,123].

Nevertheless, despite its potential, the adaptation of IB-Bio FEM as a mainstream clinical tool is still nascent, necessitating an integration of varied expertise, a topic we will delve deeper into later.

### 3.7. Advances in Biomimicry

Biomimicry, or biomimetics, refers to drawing inspiration from nature for developing new technologies and solving complex human problems. It is based on the premise that nature has already found efficient solutions to many of the challenges we face. The structure and function of natural tissues can inspire the design of orthopedic devices. For example, the lattice structure of trabecular bone or the aligned fibrous structure of tendons can guide the design of devices to foster tissue growth and integration [124,125]. Natural materials, like chitin in crustacean shells or collagen in mammalian tissues, have properties desirable in medical applications [126,127]. These materials can be mimicked or directly utilized to create biocompatible and biodegradable scaffolds. Certain organisms produce molecules that promote tissue healing, combat infections, or modulate immune responses [128,129]. By understanding and mimicking these molecules, orthopedic devices can be designed to have bioactive properties, favoring tissue integration and reducing the risk of post-operative complications. The way cells interact with their environment can be significantly influenced by the topography of the surface they are in contact with. By mimicking natural textures and structures, such as the micro-ridges found on lotus leaves or shark skin, implants can be designed to enhance specific cell behaviors or reduce bacterial colonization [130]. Some organisms possess incredible self-healing capabilities [131]. By understanding these mechanisms, researchers can design orthopedic devices that have self-repairing properties [132], increasing their lifespan and reducing the need for replacements or revisions. Some natural structures, like the responsive opening and closing of pinecones in response to humidity [133], can inspire the development of devices that adapt to their environment, responding to changes in pH, temperature, or mechanical stress. By studying how nature optimizes structures for strength, flexibility, and resilience [114,134,135], implants can be designed to better mimic the biomechanical properties of the tissues they are replacing. Some surfaces in nature, such as lotus leaves [136], have properties that prevent the adherence of contaminants [137]. This concept can be applied to orthopedic devices to prevent bacterial colonization and biofilm formation, potentially reducing the risk of implant-associated infections. Biomimicry offers a rich source of inspiration for the design of orthopedic devices, promoting their functionality, biocompatibility, and overall effectiveness.

### 3.8. Three-Dimensional Printing and Bioprinting

Three-dimensional printing technologies have profoundly driven orthopedic treatments toward personalization in several ways. Utilizing 3D-printed models based on the patient’s CT or MRI data, surgeons gain a deeper insight into the anatomy in the affected region and can anticipate potential challenges during pre-operative planning and rehearsal. With 3D printing, biomedical engineers can have an orthopedic device designed, printed, and ready for surgery in a much shorter timeframe compared to traditional methods. Three-dimensional printing technologies have not only revolutionized the fabrication of personalized orthopedic devices but also reshaped the design paradigm for these devices [138,139,140,141]. The use of 3D printing to create personalized devices based on patients’ CT or MRI data is a cutting-edge approach that has been reshaping the field of medical device design and manufacturing [142,143,144]. Traditional manufacturing techniques often produce “one-size-fits-all” orthopedic devices. Three-dimensional printing, however, allows for the creation of devices tailored to fit patients’ unique anatomy [145,146]. Three-dimensional printing can produce orthopedic devices with intricate geometries that might be difficult or impossible to achieve using conventional methods. This is especially valuable for reproducing complex anatomical structures or creating scaffolds that mimic the porous microstructure of bones [147,148,149,150]. The porosity of a scaffold plays a crucial role in nutrient diffusion, cell migration, and tissue integration. With 3D printing, the size, shape, and distribution of pores can be precisely controlled, enabling optimal cell growth and scaffold degradation rates. Multi-material 3D printing can produce orthopedic devices with varied properties in different regions [139,151,152,153], which is crucial for the device to readily integrate with the host tissue. Advanced 3D printing techniques allow for the integration of sensors and electronics directly into devices [154,155]. These can monitor various physiological parameters and provide real-time feedback, facilitating post-operative care and monitoring. Additionally, 3D-printed devices can be designed to release drugs over time in a personalized and targeted manner, enhancing therapeutic effects while potentially minimizing side effects for the patient [156,157,158,159,160].

Bioprinting takes 3D printing a step further by utilizing bioinks—materials that contain living cells. This allows for the direct fabrication of biological structures, potentially paving the way for the printing of organs or tissues for transplantation [161,162,163,164]. Bioprinted tissues can be designed using patient-specific cells [165,166,167] so that researchers and clinicians can model diseases in the lab, test potential treatments, and even design patient-specific therapies. Bioprinted tissues can be used to study drug interactions and effectiveness in a controlled environment. Furthermore, bioprinting provides researchers with the tools to create 3D cell cultures and tissue models [168,169], which can better replicate in vivo conditions compared to 2D cell cultures. This enhances the understanding of osteointegration mechanisms and the testing of potential treatments.

Three-dimensional printing and bioprinting are pioneering new frontiers in personalized orthopedic treatments, tailoring solutions to each patient’s distinct anatomy and biological requirements. With continued advancements, these technologies are poised to elevate the precision and effectiveness of orthopedic interventions, ensuring optimal compatibility and results for patients.

### 3.9. Wearable and Implantable Sensors

Wearable and implantable sensors have significant potential to revolutionize the field of personalized orthopedic treatments. These sensors can provide real-time data collection and continuous monitoring, allowing for more informed treatment decisions, proactive interventions, and better long-term patient outcomes.

Implantable sensors can provide continuous feedback on the biomechanical forces acting on devices [170,171], ensuring they remain within safe limits. Any deviation from expected behavior can be detected early, enabling timely interventions. Sensors can monitor the integration of devices with surrounding tissues [172]. For instance, changes in impedance might indicate tissue growth within a scaffold [173], which can be an essential marker for successful tissue regeneration. Implantable sensors can detect signs of infection, inflammation, or tissue necrosis, providing information for early and targeted interventions, which can improve treatment outcomes [174,175]. Sensors can gauge temperature, pH, and oxygen levels around the device, providing insights into the local cellular environment and its potential impact on device integration and functionality [176,177,178]. For devices designed to release drugs or therapeutic agents over time, sensors can monitor the local drug concentration, ensuring that therapeutic levels are maintained and adjusted as needed [179,180]. Wearable sensors can track patient movements and activities post-operatively [181,182]. These data can inform personalized rehabilitation plans, ensuring patients do not exert undue stress on the implant area and adhere to recommended activity levels. Wearable sensors can provide feedback to patients, reminding them of activity restrictions, medication schedules, or rehabilitation exercises so that they follow post-operative care instructions [183,184]. Data gathered from implantable and wearable sensors can be fed back to the design and manufacturing processes [185,186], refining and optimizing future orthopedic designs based on real-world performance. Modern sensors often come equipped with wireless data transmission capabilities [187]. This allows healthcare providers to remotely monitor the status of the device and the patient’s health, reducing the need for frequent in-person check-ups. Combined with AI and machine learning [188], data from these sensors can be used to predict potential complications or failures, facilitating preemptive interventions.

Wearable and implantable sensors offer a dynamic and responsive approach to personalized orthopedic treatments. By providing continuous data and insights, these devices empower clinicians to offer more precise, proactive, and patient-centric care, ultimately improving the success rates and longevity of orthopedic treatments.

## 4. Interdisciplinary Gaps and Bridging Strategies

The technical advancements outlined in the previous section undeniably herald a new era in personalized orthopedic treatments. Yet, bringing them to full fruition in a clinical environment is challenging, primarily due to the multidisciplinary origins of these innovations and the resultant interdisciplinary gaps. As it stands, clinicians aiming to leverage these advancements would need to acquire proficiency across multiple techniques, a task that is almost insurmountably daunting. An alternative approach, assembling a multidisciplinary team for each patient, presents financial and time constraints. In this section, we first delve into the primary interdisciplinary gaps, offering strategies to bridge them. This includes performing non-invasive quantitative characterization of the affected bone’s quality and strength, ensuring the personalized biocompatibility of device materials, and customizing device designs to address individual biological and mechanical needs. Subsequently, we outline the vision for an ideal, seamlessly integrated, and fully automated system for personalized orthopedic treatments tailored for clinician usage.

### 4.1. Non-Invasive Quantitative Characterization of Bone Quality and Strength

The success of orthopedic treatments is intimately linked to the mechanical quality and strength of the bone in question [189,190]. Bone mechanical properties form the foundation for personalized orthopedic treatments and play a pivotal role in the design and evaluation of orthopedic devices for several compelling reasons. The bone under treatment provides a foundational support for the orthopedic device. In computer-aided evaluations of device integrity and potential failure points, the mechanical attributes of the affected bone set the boundary conditions. The accuracy of simulated stresses and strains within the device can be significantly influenced by the mechanical properties of the treated bone [191]. At the heart of orthopedic treatments lies the well-being of the host bone. Any undue stress concentrations induced by the orthopedic device, which could precipitate bone damage, must be meticulously circumvented. The intricacies of the interaction between the device and the host bone are instrumental in influencing the long-term success of a treatment [192,193]. Bones are dynamic entities, perpetually remodeling in reaction to various external factors, including implants. An intimate understanding of this nuanced device–bone interplay is imperative. Ignoring this dynamic can lead to complications like implant loosening, stress shielding, or even bone resorption. It is worth noting that the well-being of the host bone and bone–device interactions are often overlooked in contemporary orthopedic device design methodologies.

In the field of mechanical and biomechanical research, the mechanical properties of bones have been extensively investigated [194,195]. The results revealed that bones have extremely complicated properties that can be possessed by any engineered or synthetic materials, including inhomogeneity, anisotropy, and visco-plasto-elasticity [196], and the properties are significantly affected by the subject’s physiological factors, such as age and gender [197]. However, the developed methodologies by mechanical engineers are often destructive, meaning they damage or alter the bone sample in the process of testing. For patients, this means that these methods cannot be applied directly to their bones without invasive procedures, making them less feasible for routine clinical assessments.

Clinicians predominantly rely on diagnostic tools like X-rays or CT scans to assess bone conditions. While these imaging techniques offer a wealth of information, they typically present a qualitative picture—highlighting bone density, structure, and potential anomalies. However, they fall short in providing precise quantitative data on the bone’s mechanical properties, such as its elasticity and strength. Biomechanical engineers have ventured to uncover the correlation between bone mechanical properties and data gleaned from medical images, particularly voxel intensity. Voxel intensity in CT scans is indeed correlated with bone density. Based on the Beer–Lambert law [198], X-rays passing through the body are absorbed to varying degrees by different tissues. Denser tissues, such as bones, absorb more X-rays and appear brighter (whiter) in the image. Bone density also influences mechanical properties like Young’s modulus and yield stress. However, the relationship between bone density and mechanical properties is not linear or consistent; materials with identical densities can exhibit stark variations in stiffness and strength. Furthermore, very few studies have reported on the relationship between voxel intensity (or mass density distribution) and the anisotropy of bone mechanical properties, a connection that is vital for understanding bone–device interactions. Hence, the inherent limitation of X-ray-based imaging technologies in deducing bone mechanical properties lies in the fact that the imaging data do not directly correlate with these properties.

Ultrasonic imaging technologies hold promise in addressing the limitations. Commonly referred to as ultrasonography, ultrasonic tomography, or ultrasound scanning, this technique utilizes mechanical sound waves, often at elevated frequencies, to depict the microstructure of materials. The method gauges the velocity of sound waves as they traverse through a medium. This speed is determined by the medium’s density and elasticity. As such, ultrasonic imaging captures data directly related to the medium’s elastic properties. With the recent advancements in this imaging technique, coupled with intricate data analysis methods, there’s now an enhanced capability to map elasticity variations within bone structures more accurately [199,200,201].

### 4.2. Patient-Specific Biocompatibility

Material biocompatibility has been recognized as the predominant factor behind the long-term failure of orthopedic treatments [202,203,204]. The human body is intrinsically programmed to reject or react adversely to foreign materials. Materials that are not biocompatible can trigger an immune response, leading to inflammation. Chronic inflammation around the implant can lead to loosening of the implant or osteolysis. For many orthopedic devices, proper integration with the surrounding bone and soft tissues is essential for long-term success. A material that is not biocompatible may not allow for this integration, leading to device failure.

Attaining optimal biocompatibility presents two main challenges: firstly, biocompatibility can vary based on individual patients, and secondly, the biocompatibility of emerging biomaterials remains less understood. Material biocompatibility can be patient-dependent. Different individuals have distinct immune systems with unique sensitivities and predispositions. What might be biocompatible for one person might trigger an allergic or immune reaction in another [205]. Genetic differences can also influence how a person’s body reacts to foreign materials. For instance, some genetic markers are associated with a higher risk of implant failure or metal allergies. Patients with certain conditions, like autoimmune disorders, diabetes, or compromised immune systems, might have a different response to implanted materials compared to healthy individuals. Depending on the patient’s body chemistry, implanted materials might degrade differently, potentially releasing byproducts that could be more reactive in some individuals than in others.

While novel biomaterials hold significant promise for advancing personalized orthopedic treatments, they also present unique issues regarding patient-specific biocompatibility [206]. While they share many of the issues encountered with conventional materials, some challenges are particularly pronounced for these new substances. Notably, many emerging biomaterials are yet to be backed by extensive long-term clinical data, making it challenging to anticipate their performance over prolonged durations and identify potential late-onset complications.

To address these challenges, the strategic approach should begin at a foundational level: understanding the nuanced interplay between a material’s chemical composition and the biological/genetic markers indicative of its biocompatibility. For a set of candidate materials, begin with a thorough analysis of the chemical composition of the materials, including any additives or contaminants; expose various cell types like osteoblasts for bone implants to each of the materials to monitor cellular responses such as adhesion, proliferation, differentiation, and viability; further, assess the cellular secretion of cytokines and growth factors to discern either inflammatory or regenerative reactions to the materials. Concurrently, establish comprehensive panels showcasing biological and genetic markers. These should include both beneficial outcomes, like tissue integration and regeneration, and undesirable reactions, such as inflammation or tissue death. To delve deeper into material biocompatibility, employ in vitro genetic and proteomic studies. Expose human cell lines to individual materials and then extract RNA. Utilize techniques such as qRT-PCR or RNA sequencing to investigate any shifts in gene expression upon material exposure. Similarly, analyze protein extractions to see how these materials affect protein expression patterns.

The massive data gathered from these methods can then feed into an AI-driven system, which will evolve with every new set of data, including results from clinical trials. This sophisticated AI platform holds the promise of predicting a material’s biocompatibility for a specific patient using only the material’s chemical composition and the patient’s unique biological and genetic markers.

### 4.3. Personalized Design of Orthopedic Devices with Optimal Balance between Biological and Mechanical Requirements

**Figure 2:**
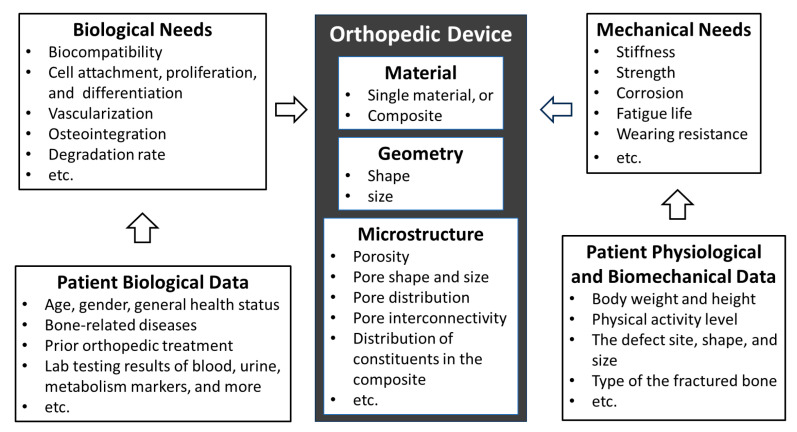
Factors and variables to consider in the design of orthopedic devices.

For the successful long-term performance of orthopedic devices, it is essential that the design address both mechanical and biological demands, as depicted in Figure 2. Historically, the orthopedic sector evolved with a primary focus on addressing fractures, deformities, and mechanical disorders. As such, the contemporary design of orthopedic devices predominantly focuses on mechanical factors [207] such as geometric shape, size, stiffness, strength, corrosion, etc. The biological needs have been mostly overlooked. While the biocompatibility of materials is a foundational requirement for the device, there are many other biological needs to meet. To seamlessly integrate with the host bone, an orthopedic device must support cellular attachment, proliferation, differentiation, and vascularization. Moreover, it should have an appropriate degradation rate to match the natural healing or regeneration process. These biological performances are significantly influenced by the device’s microstructure, which encompasses features like surface roughness, porosity, pore shape and size, interconnectivity, and pore distribution. The primary challenge in achieving an optimal design for a personalized orthopedic device does not just stem from the large number of biological and mechanical variables but also from the fact that these variables are patient-specific. Furthermore, these factors often interact with or counteract one another, making the design process a complex balancing act.

Regarding the patient-dependent nature of the variables, in the preceding section, we emphasized that biocompatibility is unique to each individual, largely addressed by choosing the right material for the device. Figure 2 further highlights other biological factors that can vary from patient to patient. The device’s interaction with cellular activities, including attachment, proliferation, differentiation, and vascularization, is significantly influenced by the patient’s age, gender, genetic profile, overall health, and any existing bone-related conditions. On the mechanical side, considerations such as stiffness, strength, toughness, wear resistance, and fatigue resistance are intimately tied to the patient’s physical attributes, like body weight and height. They also relate to the specific functions and characteristics of the affected bone, including its location and type, and the patient’s physical activity level.

There are numerous potential interplays and counterplays between the biological and mechanical requirements. A few examples are provided below. Some materials that offer excellent mechanical properties might not be biocompatible. Conversely, biocompatible materials might not always provide the desired mechanical strength or durability. Materials that are too stiff may lead to stress shielding, where the device supports most of the mechanical load, causing the surrounding bone to atrophy from disuse. This can undermine osteointegration, as the bone might resorb or weaken around the implant. For joint orthopedic devices, a roughened surface might promote cell attachment and proliferation. However, increased surface roughness can reduce wear resistance, leading to premature device failure or the generation of wear particles that can cause inflammatory reactions. Orthopedic devices made of biodegradable materials are designed to be incrementally substituted by native tissue. It is crucial that their degradation rate align with the pace of tissue regeneration. If not, the materials might weaken prematurely, compromising the device’s stability. A high-porosity microstructure can enhance vascularization and nutrient delivery, promoting tissue integration. However, increasing the porosity can compromise mechanical strength and stiffness. Materials that promote cell differentiation, especially toward an osteogenic lineage, are ideal. However, the same materials might be susceptible to corrosion, which can release toxic ions or create a less favorable environment for cell differentiation and function. Surface coatings or treatments can improve the corrosion resistance of metals but can also reduce their bioactivity. The mechanical environment, such as the stresses and strains applied to the device, can influence cellular activities within and surrounding the device. Appropriate mechanical loading can enhance osteogenesis, but excessive or inadequate loading can deter it.

For the optimal design of orthopedic devices, considering the large number of variables that are patient-specific and can intricately interact with or counteract each other, the traditional trial-and-error approach is impractical or, at the very least, extremely time-consuming. Leveraging multidisciplinary computer modeling may present a more efficient solution [208,209]. Multidisciplinary computer modeling combines both engineering and biological methodologies to comprehensively address the challenges in designing orthopedic devices. On the one hand, engineering modeling methods, like the finite element method (FEM), play a crucial role in assessing the mechanical properties, stress distribution, and overall structural integrity of the device under varying conditions. The FEM enables the prediction of how the mechanical behavior of the device, such as stiffness and strength, will change with the device geometry, material, and microstructure, providing an efficient way to validate or exclude device designs. On the other hand, biological modeling methods delve into the patient-specific physiological interactions that the device will encounter once implanted. These models simulate tissue growth, inflammation, and the integration process of the device with the surrounding biological environment. By doing so, they help in predicting biocompatibility, ensuring that the device promotes optimal healing and tissue regeneration without adverse reactions. Together, these intertwined modeling methods offer a comprehensive solution, allowing for the simultaneous optimization of both mechanical and biological factors in orthopedic device design. This synergy ensures that devices are not only structurally sound but also harmoniously integrated into the body, leading to the best possible outcomes for patients.

### 4.4. Seamless Integration of Multidisciplinary Technologies and Full Automation of Personalized Orthopedic Treatment

The motivation to integrate and automate emerging technologies across disciplines stems from the ambition to empower clinicians to use these technologies independently. Recognizing the multidisciplinary nature of these technologies, it is impractical and daunting to expect clinicians to become adept in every aspect of the technologies, especially given their already extensive training regimen. Currently, a prevalent strategy involves assembling a specialized multidisciplinary team tailored to each patient’s needs whenever such technologies are integral to the treatment. However, this approach can be both resource-intensive and time-intensive. Furthermore, assembling such teams is not always feasible due to the availability of specialists.

**Figure 3:**
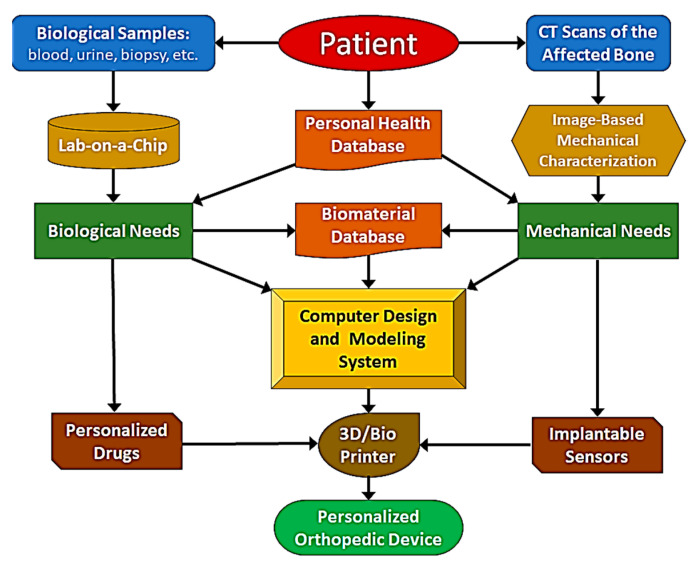
Intelligent system for personalized orthopedic treatments.

An ideal solution would be creating an intelligent system that seamlessly integrates and fully automates these diverse technologies. As depicted in Figure 3, this system is intended for use by clinicians without necessitating the involvement of an entire team of experts. Central to this system is a robust interconnected infrastructure, granting instantaneous access to pivotal databases, potent computational capabilities, and innovative decision-making algorithms honed for individualized treatments. This system would manage the entire workflow of an orthopedic treatment, from the initial diagnosis to the bedside delivery of a fully personalized orthopedic device. Key components of this infrastructure would include a comprehensive personal health record database, specialized medical imaging units, a lab-on-a-chip setup, a repository of biomaterials, cutting-edge computing systems equipped with computer-aided design and modeling software, and state-of-the-art 3D and bioprinting devices. As illustrated in Figure 3, the workflow would start with the patient’s biospecimens (blood, urine, biopsy, etc.) and diagnostic imaging scans of the affected bone, and the output would be a fully personalized device delivered to the patient’s bedside for implantation. This automation could be taken a step further; a networked 3D printing/bioprinter on the patient’s bedside could potentially fabricate the personalized device directly into the patient’s body.

Both bridging the interdisciplinary gaps and developing the intelligent system require collaborative endeavors that stretch beyond conventional spheres of expertise. Such endeavors mandate a robust multidisciplinary collaboration. Clinicians, at the forefront of patient care, provide crucial insights into patient demands and the complexities of orthopedic treatments. Their firsthand experience with patients aids in shaping the design and functionality of the intelligent system. Biomechanical and biomedical engineers play a pivotal role by translating these clinical insights into tangible devices and systems. Their expertise ensures that the intelligent system is not only reliable and effective but also ergonomic and user-friendly.

The development and continuous updating of the biomaterial database require concerted efforts from biologists, chemists, material scientists, and clinicians. Biologists contribute by understanding the intricate cellular and tissue interactions with the biomaterials, ensuring that the treatments are biocompatible and promote optimal healing. Chemists, on the other hand, can aid in refining materials and coatings that are used in devices, making them safe, durable, and responsive to the body’s environment. Material scientists delve into the nitty-gritty of device composition, exploring innovative materials that offer the desired balance between durability, biocompatibility, and adaptability. Clinicians can provide real-world feedback on how well the biomaterials interact with the body, any adverse reactions, or longer-term complications. Their work ensures that the biomaterials are not just functional but also long-lasting and safe for application in any individual.

Last but certainly not least, AI researchers and engineers can play an indispensable role, serving as the linchpin that seamlessly weaves together the myriad technological components of the system. By developing sophisticated algorithms and machine learning methodologies, they infuse the system with advanced intelligence. This capability allows the system to preemptively select the most appropriate one among available options at each step.

The suggested strategies and solutions in this section are tentative and serve as a starting point for addressing the interdisciplinary gaps. We recognize the need for more specific details and examples of successful interdisciplinary collaboration. Future work will focus on developing a detailed roadmap outlining the steps needed to achieve full automation in personalized orthopedic treatments.

## 5. Conclusions

The landscape of orthopedic treatments has seen significant evolution over recent years, with a clear inclination toward personalization to enhance long-term success. It is evident that groundbreaking advancements across multiple disciplines are continually reshaping the domain of orthopedics. Each technological advancement discussed in this review—be it artificial intelligence, biomaterials, genomic and proteomic analyses, lab-on-a-chip, medical imaging, image-based biomechanical finite element modeling, biomimicry, 3D printing and bioprinting, or implantable sensors—holds immense potential in the quest for refined personalized orthopedic treatments. However, the primary beneficiaries of these advancements, the clinicians, find themselves in a quandary. The rapid pace of technological evolution demands either perpetual upskilling to keep abreast of all advancements or the assembly of a bespoke multidisciplinary team for each patient—both approaches being less than optimal.

The solution to this conundrum lies in crafting an intelligent system that seamlessly integrates and fully automates these diverse technologies. Such a system would empower clinicians to merely input patient diagnostic details and health records and, in return, receive a personalized orthopedic device. However, the path to achieving such an integrated and automated platform is riddled with interdisciplinary gaps. These include the non-invasive quantitative assessment of bone quality and strength, the consideration of patient-specific biocompatibility, and personalized device design with a balance between biological and mechanical needs. To bring this visionary system to fruition, a synergistic collaboration of experts across various disciplines—including biologists, chemists, materials scientists, biomechanical and biomedical researchers, and clinicians—is not just a desire but a necessity.

## Data Availability

Not applicable.
